# Analysis of the changing trend of economic burden of patients with chronic diseases under the Integrated Medical and Health Service System

**DOI:** 10.1186/s12889-023-15572-4

**Published:** 2023-04-21

**Authors:** Xuedan Pei, Bing Wang, Xiaohong Yang, Wenming Feng, Yanqiu Sun, Hao Wang, Li Gao, Yinuo Gu, Yening Bei

**Affiliations:** 1 Tongshan District Jifu Hospital, Xuzhou, 221112 China; 2 Huzhou University, Huzhou, 313000 China; 3 Huzhou First People’s Hospital, Huzhou, 313000 China; 4 Xuzhou Oriental People’s Hospital, Xuzhou, 221004 China

**Keywords:** Medical and health service system, Chronic diseases, Economic burden, Change trend

## Abstract

**Background:**

Integrating medical resources is one of the explorations of medical mechanism reform to meet the needs of whole-cycle health management and is an important initiative in the current round of China's healthcare system reform. 2015 saw the construction of county medical communities to promote the balanced layout of medical resources, which opened a new exploration of the construction of an integrated healthcare service system in China. 2017 saw the promotion of the pilot construction of compact county medical communities in Zhejiang Province, China.

**Objective:**

From the perspective of alleviating the financial burden on those in need of health services, the characteristics of chronic disease patients' access to health care and the composition and changing curve of the medical cost burden are analyzed to provide a basis for the construction path of an integrated health care service system.

**Methods:**

A retrospective cohort study was conducted to select 5739 permanent residents who met the inclusion and exclusion criteria in Z town, H city, Zhejiang province. This population's health insurance utilization data from 2015 to 2018 were retrieved, and their average annual costs, cost composition, and health insurance payments were analyzed.

**Results:**

The average annual growth rates of medical insurance and out-of-pocket costs before and after the implementation of the Medical Community were 12.85% and 9.72%, respectively. The increase narrowed significantly after the construction of the Medical Community, with the ringgit growth rate dropping to 2.73% in 2018. The top three medical expenses that accounted for the highest percentage were drug, consultation, and treatment fees. The frequency of visits to primary health care consulting hospitals has increased yearly.

**Conclusions:**

By implementing various measures to strengthen the grassroots level, patients' choice of primary care has increased year by year in the early stages of the construction of the Medical Community. From the perspective of cost control, strengthening the regulation of drugs and tests and restricting the use of high-value consumables can further reduce medical costs and ease their financial burden.

The basic medical insurance system is a universal medical insurance system in China, with basic medical insurance as the mainstay, medical assistance as the backbone, and major medical insurance as an extension, whose main purpose is to alleviate the financial burden of medical expenses on citizens after receiving treatment for illnesses [1]. After years of reform, China's medical security system has formed a medical security system consisting of the basic medical insurance for urban workers, the basic medical insurance for urban and rural residents and the new rural cooperative medical insurance system, and covers the urban employed population, the urban non-employed population and the rural population respectively, in order to achieve the goal of full medical insurance coverage. By the end of 2021, the number of people covered by basic medical insurance reached 136,424,000 [2]. In Zhejiang province, where the economy is more developed, two types of medical insurance systems have been implemented throughout the province, namely basic medical insurance for urban workers and basic medical insurance for urban and rural residents [2, 3].

The increase in the elderly population and the emergence of chronic diseases as a significant human health problem require a shift in health services from a "cure-centered" approach to a "health-centered" system based on building primary health care and health intelligence based on population density. The current hospital hierarchy in China is tertiary hospitals—secondary hospitals—primary hospitals (township health centers and community health service centers), of which township health centers/community health service centers, as an essential part of China's primary healthcare service system, are mainly responsible for meeting the immediate healthcare needs of residents and play a crucial role in promoting the construction of a healthy China and providing accessible healthcare services to the public. However, there is a wide disparity in healthcare services in China between urban and rural areas, with quality healthcare resources mainly concentrated in large hospitals in big cities. The capacity of healthcare services within counties, especially at the grassroots level, is weak. The poor service capacity of primary healthcare institutions and the residents' lack of recognition and trust are indisputable facts. As a result, the country is again reforming its healthcare system, focusing on integrating healthcare resources—the Integrated Medical and Health Service System. One of the construction aims is graded health management and orderly access to health care. One of the construction paths is to increase the capacity building of primary health care institutions; to increase the proportion of medical reimbursement for residents to seek medical treatment in primary health care institutions to guide residents to seek medical treatment in primary health care institutions more often. One of the indicators of the construction is the reduction of residents' access to primary health care institutions and the per capita disease burden. China's Anhui, Shanxi, and Zhejiang provinces have explored integrated health service systems in the form of tightly knit medical alliances and county medical communities [4]. In 2013, Zhejiang Province began to implement a "double sinking and two upgrades" policy to promote the sinking of high-quality urban medical resources and the deployment of medical personnel to the grassroots level [5]. 2017 saw the construction of county-level medical communities to promote the balanced layout of medical resources and build a high-quality and efficient integrated medical and health service system [6]. 2019 saw the Zhejiang Province Pilot construction of tightly-knit county medical communities would be promoted in the whole province [7].

To demonstrate that the Integrated Medical and Health Service System achieves primary diagnosis and reduces patients' economic burden, this study takes chronic disease patients in Z town, H city, Zhejiang province as the research object and compares 2015 and 2016 before the construction of the medical community as the control and 2017 and 2018 at the beginning of the building, and continuously tracks their medical consultation behavior and medical cost composition for four years to analyze the impact of the medical community, a The study will examine the effect of the health care service platform on chronic disease patients' health care behavior and the trend of changes in their financial burden, as well as the composition of medical costs at all levels of hospitals, to provide data support for the implementation path of achieving primary care for chronic disease patients and improving quality and reducing costs.

## Materials and methods

### Study population

A retrospective cohort study was conducted to select 5,739 residents of Z town, H city, Zhejiang province, who met the criteria for nadir and to retrieve this population's 2015–2018 medical insurance usage data. (Since basic medical insurance reimburses patients for most of their medical expenses, Chinese patients with chronic diseases register their medical insurance accounts when they visit any medical institution, which is the basis for ensuring that the medical costs of the subjects in this study are complete and accurate.). Inclusion criteria: residents with chronic diseases with four consecutive years of medical insurance usage records from 2015–2018. Exclusion criteria: incomplete information. All costs were benchmarked against 2018 data and discounted according to the Consumer Price Index (CPI) for the healthcare category [8].

### Statistical analysis

General statistical description using Excel 2019 and R 4.0.3 statistical software. The normality of the data was tested using the Shapiro–Wilk test; continuous variables with a normal distribution were expressed as mean and standard deviation [mean (S.D.)]; their per capita medical costs (cost components), per capita medical insurance costs, per capita out-of-pocket costs, and trends in per capita medical insurance costs and per capita out-of-pocket costs were analyzed using general descriptions. (Based on professional knowledge, we believe that chronic diseases, if managed properly, should be relatively stable in terms of frequency of visits and purpose of visits per year and that if extreme cost situations occur, they are due to the occurrence of essential complications or other major health events that endanger health. Therefore total costs are standardized in this paper, and data exceeding three times the standard deviation are not analyzed.)

## Results

### Population profile

A total of 5739 patients with chronic disease were included in this study, 2564 (44.7%) males and 3175 (55.3%) females, with an age range of 36–102 years and non-normally distributed data for age, described using a median and interquartile spacing of 73.00 (13.00) years.

#### Distribution of health care costs per capita by hospital level, 2015–2018

The total medical expenses for 2015–2018 were 397.32 (20.89%), 492.52 (22.84%), 622.66 (23.73%), and 699.45 (24.91%) million Yuan for first-level hospitals; the total medical expenses of the secondary hospitals were 455.98 (23.97%), 487.47 (22.60%), 578.99 (22.06%) and 608.41 (21.67%) million Yuan respectively; the total medical expenses of the tertiary hospitals were 1032.23 (54.27%), 1145.35 (53.11%), 1388.25 ( 52.90%), 1454.32 (51.79%) million Yuan; total medical expenses of pharmacies were 8.24 (0.43%), 7.46 (0.35%), 10.53 ( 0.40%), and 7.32 (0.26%) million Yuan. Table 1 shows the per capita medical costs at all levels of hospital. The year-on-year growth rates of medical expenses per capita in primary hospitals were 22.42%, 25.36%, and 9.32%, respectively; the year-on-year growth rates of medical expenses per capita in secondary hospitals were 17.10%, 21.38%, and 9.98% respectively; the year-on-year growth rates of medical expenses per capita in tertiary hospitals were 2.62%, 17.81%, and 2.36% respectively; the year-on-year growth rates of medical expenses per capita in pharmacies were -2.47%, 25.74% and -27.56% respectively. Regarding per capita medical expenses, the medical costs of primary, secondary, and tertiary hospitals are on a year-on-year upward trend. Still, since the construction of the Medical Community in 2017, the year-on-year growth rate of per capita medical costs of primary, secondary, and tertiary hospitals has been on a downward trend and has more than doubled; the year-on-year growth rate of per capita medical costs of pharmacies has been on a downward trend. In terms of the distribution of total healthcare costs across all levels of hospitals, Table 2 shows that the proportion of outpatient costs in primary hospitals is on the rise; conversely, the proportion of outpatient costs in secondary and tertiary hospitals is on the decline, with primary hospitals having the highest proportion of outpatient costs and tertiary hospitals still having a higher proportion of outpatient costs than secondary hospitals. In terms of the proportion of inpatient costs, tertiary hospitals have the highest proportion of inpatient costs, with a fluctuating downward trend; secondary hospitals are in second place, on an upward trend year on year; and primary hospitals show a fluctuating upward trend, while purchases at pharmacies are also decreasing year on year.

**Table 1:** Per capita health care costs at all levelsof the hospital (RMB), 2015–2018

Year	First-class hospital	Secondary Hospital	Tertiary Hospital	Chemist’s shop
2015	756.36	1417.42	5274.67	493.54
2016	925.97	1659.75	5412.79	481.35
2017	1160.81	2014.58	6377.02	605.26
2018	1268.95	2215.62	6527.47	438.47

**Table 2:** Distribution of outpatient and inpatient health care costs by level of the hospital (million Yuan), 2015–2018

Year	Primary Hospital policlinic be in the hospital		Secondary Hospital policlinic be in the hospital		Tertiary Hospital policlinic be in the hospital		Chemist’s Shop policinic buy medical	
2015	38.67	0.28	30.92	14.86	32.72	70.50	0.02	0.00
2016	48.24	1.02	30.89	17.86	38.16	76.38	0.00	0.74
2017	55.19	7.08	31.12	26.78	41.41	97.41	0.00	1.05
2018	63.92	6.03	28.90	31.95	42.49	102.94	0.00	0.73

#### Composition of medical costs per capita at all levels of the hospital, 2015–2018

The top three highest medical costs per capita in primary hospital were drug, consultation and treatment fees, followed by examination and consumables; the top three highest medical costs per capita in secondary hospital were drug, examination and treatment fees, followed by consumables and consultation; the highest medical costs per capita in tertiary hospital were drug, examination and consumables, followed by treatment and surgery; the per capita drug The cost per capita of medicine is on a year-on-year rising trend, while its per capita consultation and treatment cost in 2018 has a year-on-year growth rate of 43.53%, which is more than the 0.91% of Secondary Hospital and 0.64% of Tertiary Hospital; the highest ranking of examination and treatment cost per capita is: Tertiary Hospital, whose per capita examination and treatment cost has exceeded RMB1,000; the per capita cost of consumables at all levels of hospitals is on a rising trend, in 2018 annual growth rates of 59.99%, 33.29% and 6.82% respectively.

#### Trends in medical insurance costs, out-of-pocket costs, 2015–2018

**Fig. 1:**
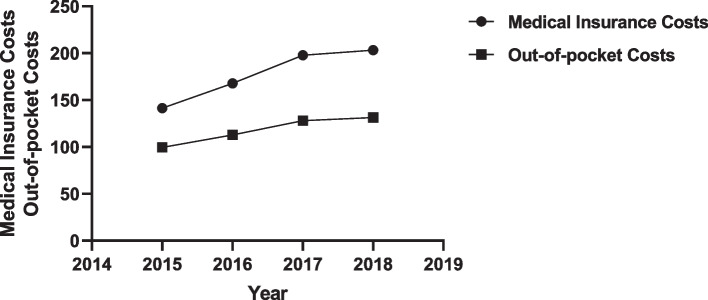
Change trend of Medical Insurance Costs and Out-of-pocket Costs from 2015–2018

The 2015–2018 medical insurance costs were RMB141.41, RMB167.75, RMB197.81, and RMB203.21, respectively, with an average annual increase of 12.85% and year-over-year growth rates of 18.63%, 17.92%, and 2.73%, respectively; the 2015–2018 out-of-pocket spendings were RMB99.38, RMB112.87, RMB128.05, and RMB131.26, with an average annual increase of 9.72% and annual growth rates of 13.49%, 15.18%, and 3.21%, respectively, on a chain basis. As is showed in Fig. 1, the medical insurance and out-of-pocket spending for chronic diseases and their co-morbidities under the medical association is rising.

## Discussion

### Patients' choice of primary care has increased year after year in the early days of the Medical Community

China's three-tier healthcare system is led by tertiary hospitals, which bring together county hospitals and township health centers (street community health centers) in their regions to provide medical treatment. Zhejiang launched the pilot construction of the Medical Community in 2017, and this study shows (Tables 1 and 2) that under the role of the Medical Community, patients with chronic diseases have gradually shifted to the primary medical and health institutions; patients' self-health awareness has increased and they no longer use medication blindly. However, as far as the distribution of medical costs among all levels of hospitals is concerned, the medical costs of tertiary hospitals are still higher. Although the medical costs of primary and secondary hospitals are increasing, the increase is still lower than that of tertiary hospitals. County hospitals should give full play to their leading role in driving community medical institutions in the county, improve their business standards and service capabilities, direct patients to community medical institutions, upgrades the degree of satisfaction of patients, and promote the formation of a clear functional, and efficient healthcare service system and a graded treatment model with primary care, two-way referral, acute and slow treatment, and upward and downward linkage. As seen in Fig. 1, the medical costs and out-of-pocket expenses of patients with chronic diseases are all on the rise, but their year-on-year growth rates are all on the decline, and the rate of decline is more than 10%. The data shows that the financial burden on patients with chronic diseases has been significantly reduced in the early stage of the construction of the medical community, and the trend of patients choosing primary medical and health institutions are growing. In this context, the health insurance sector can further explore health insurance suitable for the medical community model and establish a health insurance system suitable for chronic diseases and their co-morbidities [9], especially concerning the reimbursement treatment of general urban and rural residents' health insurance [10], to further promote patients to seek health services in primary care institutions through economic leverage in order to lower their out-of-pocket expenses and reduce their financial burden.

### Medical insurance departments should strengthen the regulation of drug and examination costs and limit the use of high-value consumables

The primary purpose of building and developing a Medical Community is to play the leading role of tertiary public hospitals in the region and to guide different levels and types of medical institutions to establish a clear division of labor and collaboration with clear objectives, powers, and responsibilities [11]. As can be seen from Table 3, the construction of the Medical Community has so far gradually completed the work of positioning and dividing the work between the various levels of health care institutions, with primary medical and health institutions beginning to take on the prevention, treatment, and management of chronic diseases, and tertiary hospitals begin to devote in providing a higher level of care for patients. Excluding the cost of medical services (diagnosis and treatment fees), this study shows that the per capita cost of drugs is the largest, followed by the cost of examination and material. After the implementation of the zero-percentage increase in drug costs, drug costs have shifted more to examination and material costs, especially in tertiary hospitals, where the per capita examination costs have exceeded RMB 1,000 and are still on the rise, with the most significant increase in examination costs in secondary hospitals, which are higher than those in tertiary hospitals; the per capita consumables costs in all levels of hospitals are on the rise, with tertiary hospitals having the highest percapita material costs, the reason for which may be related to the level of tertiary hospitals and their ability to undertake higher levels of treatment and surgery. The results of this study are consistent with the findings of Zhou Minghua [12] and Zhao Li [13]. Therefore, the health insurance department should strengthen its supervision in terms of drug prescriptions and medication levels to reduce the number of times and the number unnecessary drugs used, thus reasonably controlling the growth of drug revenues [14]; further promoting the mutual recognition system of test and examination results, regulate doctors' treatment behaviors and minimize unnecessary tests and duplication [15], and unified examination fees can be set to reduce examination costs. Restrict the use of high-value material, and suggest that the medical insurance department take the lead in organizing the procurement of high-value medical consumables concerning the model of drug procurement with volume to significantly reduce the procurement cost of high-value consumables thus reducing medical costs and patient fees [16].

**Table 3:** Composition of health care costs per capita by the level of hospital

Year	First-class hospital						Secondary Hospital						Tertiary Hospital					
	Drug	Diagnosis	Examine	Treatment	Material	Own expense	Drug	Diagnosis	Examine	Treatment	Material	Own expense	Drug	Diagnosis	Examine	Treatment	Material	Own expense
2015	668.19	9.35	8.15	18.09	2.58	0.00	860.20	58.35	270.36	148.17	80.08	0.25	2009.43	92.17	1277.49	1065.67	763.47	66.45
2016	829.62	55.44	14.17	24.27	2.38	0.00	952.56	57.66	321.25	213.79	114.34	0.16	2102.67	94.05	1365.65	997.90	826.68	25.84
2017	971.72	55.40	33.68	80.80	19.22	0.00	1085.62	62.37	399.22	295.85	162.47	9.05	2311.53	101.78	1552.27	235.45	1125.54	50.32
2018	1069.73	79.52	33.20	57.71	30.75	0.03	1005.78	62.94	529.22	400.86	216.56	0.25	2223.69	102.43	1574.42	1391.91	1202.30	32.72

### Limitations

This study only used health insurance data from Z town in H city, Zhejiang province, which has some geographical limitations; we only analyzed health insurance data from 2015–2018, a short period after the construction of the integrated health care service system, and only explored the result that the growth rate of health insurance costs and out-of-pocket costs decreased, which did not achieve our expected goal—that is, the integrated health care service system We did not reach our desired goal of reducing both health insurance costs and out-of-pocket costs after the implementation of the integrated health care delivery system. We will continue this study in the future; this study is based on the fact that residents use their health insurance accounts to access health care, but in practice, there are some cases where residents purchase health care services on their own through out-of-pocket payments, which will affect the accuracy of the data in this study.

## Conclusion

Our research shows that the integrated health service system has gradually completed the positioning and division of labor between the different levels of healthcare institutions, with primary healthcare institutions taking on the prevention, treatment, and management of chronic diseases and tertiary hospitals providing a higher level of care for patients. In terms of reducing the financial burden, although health insurance costs and out-of-pocket expenses are rising, their year-on-year growth rates are declining and have fallen by more than 10%. Over time, we believe that health insurance and out-of-pocket costs will trend downwards and that integrated health services will significantly contribute to reducing the financial burden on patients with chronic diseases.

## Data Availability

This dataset isn’t publicly available. To request data for this study, please get in touch with the corresponding author: Bing Wang, at 00909@zjhu.edu.cn.
